# Arc-driven mGRASP highlights CA1 to CA3 synaptic engrams

**DOI:** 10.3389/fnbeh.2022.1072571

**Published:** 2023-01-30

**Authors:** B. K. B. Murthy, S. Somatakis, A. F. Ulivi, H. Klimmt, T. P. Castello-Waldow, N. Haynes, R. E. Huettl, A. Chen, Alessio Attardo

**Affiliations:** ^1^Leibniz Institute for Neurobiology, Magdeburg, Germany; ^2^Graduate School of Systemic Neurosciences, Munich, Germany; ^3^Max Planck Institute of Psychiatry, Munich, Germany; ^4^International Max Planck Research School for Translational Psychiatry, Munich, Germany; ^5^Weizmann Institute of Science, Rehovot, Israel

**Keywords:** synaptic engram, dorsal hippocampus, fear conditioning, mGRASP, cellular engram

## Abstract

Subpopulations of neurons display increased activity during memory encoding and manipulating the activity of these neurons can induce artificial formation or erasure of memories. Thus, these neurons are thought to be cellular engrams. Moreover, correlated activity between pre- and postsynaptic engram neurons is thought to lead to strengthening of their synaptic connections, thus increasing the probability of neural activity patterns occurring during encoding to reoccur at recall. Therefore, synapses between engram neurons can also be considered as a substrate of memory, or a synaptic engram. One can label synaptic engrams by targeting two complementary, non-fluorescent, synapse-targeted GFP fragments separately to the pre- and postsynaptic compartment of engram neurons; the two GFP fragments reconstitute a fluorescent GFP at the synaptic cleft between the engram neurons, thereby highlighting synaptic engrams. In this work we explored a transsynaptic GFP reconstitution system (mGRASP) to label synaptic engrams between hippocampal CA1 and CA3 engram neurons identified by different Immediate-Early Genes: *cFos* and *Arc*. We characterized the expression of the cellular and synaptic labels of the mGRASP system upon exposure to a novel environment or learning of a hippocampal-dependent memory task. We found that mGRASP under the control of transgenic ArcCre^ERT2^ labeled synaptic engrams more efficiently than when controlled by viral cFostTA, possibly due to differences in the genetic systems rather than the specific IEG promoters.

## Introduction

Memories are thought to be encoded as enduring physical changes in the brain. In fact, in murine models, not only subpopulations of neurons throughout various brain regions show increased neuronal activity during memory formation, but manipulation of the activity of these neurons can induce artificial retrieval or loss of stored memories (Zhou et al., 2009; Liu et al., 2012; Ramirez et al., 2013; Denny et al., 2014; Tanaka et al., 2014a; Vetere et al., 2017; Josselyn and Tonegawa, 2020). This demonstrates that memory storage and retrieval are mediated by subpopulations of neurons which are thus believed to be cellular engrams. According to the Hebbian postulate, connections between neurons with correlated activity patterns are strengthened while connections between neurons whose activity patterns are weakly correlated are depressed or even lost (Hebb, 1949). This phenomenon increases the probability of neural activity patterns occurring during encoding to re-occur at later time points. Therefore, the subset of synapses between coactive neurons can also be considered as a substrate of memory, or a synaptic engram. While an ever-increasing number of studies investigates engrams at the system and circuit levels (Reijmers et al., 2007; Liu et al., 2012; Ramirez et al., 2013, 2015; Denny et al., 2014; Hsiang et al., 2014; Kawashima et al., 2014; Redondo et al., 2014; Tanaka et al., 2014b; Rashid et al., 2016; Kitamura et al., 2017; Vetere et al., 2017; Rao-Ruiz et al., 2019; Visser et al., 2020; Domi et al., 2021), studies focusing on the synaptic level are scarce. This is because investigating the activity of defined synapses and tracking the changes in these synapses through time is technically challenging. Thus, it remains unclear whether memory formation truly enhances synapses between neurons of connected brain regions.

Studying the stability of structural synaptic connectivity as a proxy for strength of synaptic activity helps circumvent this problem. Stability of dendritic spines in rodents is associated with memory formation and recall (Trachtenberg et al., 2002; Zuo et al., 2005a,b; Xu et al., 2009; Yang et al., 2009, 2016; Fu et al., 2012; Attardo et al., 2015; Castello-Waldow et al., 2020; Chenani et al., 2022; Gallinaro et al., 2022) and increasing stability of neocortical dendritic spines enhances learning while decreasing the size of neocortical dendritic spines leads to impaired performance in a motor task (Hayashi-Takagi et al., 2015; Albarran et al., 2021). Recently, it has become possible to label synapses between neurons, thanks to the GFP Reconstitution Across Synaptic Partners [GRASP (Feinberg et al., 2008)] and its homologous optimized for mammalian expression [mGRASP (Kim et al., 2011; Druckmann et al., 2014)] techniques. Both GRASP and mGRASP use two complementary, non-fluorescent GFP fragments, which are expressed separately on pre- and postsynaptic membranes and reconstitute in the synaptic cleft to form functional GFP, thus pinpointing synapses between specific pre- and postsynaptic neurons. More recently, an enhanced version of the mGRASP system (eGRASP) was targeted to pre- and postsynaptic engram neurons with a genetic method based on the Immediate Early Gene (IEG) *cFos* (Reijmers et al., 2007) and enabled to visualize the engram at the synaptic level (Choi et al., 2018, 2021; Choi and Kaang, 2022). These advancements could enable to visualize synaptic engrams in the live mouse by using intravital two-photon microscopy and the mGRASP system seems to be better suited to this aim given the lower number of fluorescent proteins involved and their better spectral separation. We thus decide to test whether the mGRASP system could also be used to label synaptic engrams when driven by the IEG *cFos*. The genetic system based on the IEG *cFos*, however, provides only transient labeling and it is unclear whether other genetic schemes based on other IEGs (Kawashima et al., 2009, 2013; Guenthner et al., 2013; Sørensen et al., 2016), could improve labeling of structural synaptic engrams. We thus also investigated the use of mGRASP system in the dorsal hippocampus of mice under the control of two different IEG promotors commonly used to identify neuronal engrams: *cFos* and *Arc*. We marked *cFos*-expressing cells by using Adeno-Associated Viruses (AAVs) in which the *cFos* promoter drives the transcription of a tetracycline Trans Activator (tTA) (Zhang et al., 2015), which in turn drives the transcription of mGRASP. To detect *Arc*-expressing cells we employed a transgenic mouse line in which the endogenous *Arc* promoter drives the transcription of a Cre recombinase whose activity is gated by Tamoxifen (Cre^ERT2^) (Guenthner et al., 2013), which in turn drives the transcription of mGRASP. We then characterized the expression of the cellular and synaptic labels of the mGRASP system upon exposure to an Enriched Environment (EE) or upon learning of the hippocampal-dependent memory task Trace Fear Conditioning (TFC). mGRASP under the control of transgenic ArcCre^ERT2^ labeled synapses between CA1 and CA3 pyramidal neurons active during EE and TFC more efficiently than when controlled by viral cFostTA. However, we think this difference reflects the difference between the genetic systems we employ rather than between the IEG promoters.

## Materials and methods

### Subjects

Animals were C57Bl6/N bred in house (for cFostTA-dependent expression), ArcCre^ERT2^-Ai9 double transgenic or ArcCre^ERT2^ single transgenic on C57Bl6/N background (for ArcCre^ERT2^-dependent expression) male and female mice between 3 and 6 months of age with free access to food and water and a 12/12 light/dark cycle. All animal procedures conformed to the guidelines of the Max Planck Society and the local animal authority (Regierung von Oberbayern – Veterinärwesen) and were approved in the License for animal experimentation # ROB-55.2Vet-2532.Vet_02-17-150.

### Viral injections

Intracranial injections of Adeno Associated Viral suspensions were carried out according to standard methods. We injected 200–400 nL of a viral suspension (see Supplementary Table 1 for details about viruses) at a rate of 100 nL/min in dCA1 (AP, –2.0 mm; ML, 1.4; DV, 1.4 mm) or in dCA3 (AP, –2.0 mm; ML, 2.2; DV, 2.2 mm). Mice were allowed to recover for a minimum of 10 days.

### Plasmid and virus production

To produce the TRE-pre-mGRASP construct, an AAV vector backbone with the TRE promoter was obtained from pAAV-TRE-tdTomato-WPRE (#104112, Addgene) *via Eco*RI and *Hin*dIII digestion. The pre-mGRASP-mCerulean encoding sequence was amplified from pAAV-CAG-pre-mGRASP-mCerulean (#34910, Addgene) by polymerase chain reaction (PCR) using two oligonucleotide primers containing *Eco*RI and *Hin*dIII restriction sites (5′-caagaattcATGCCACCTTCTACTAGTC-3′ and 5′- cacaagcttTCACTTGTACAGCTCATC-3′) and inserted into the pAAV-TRE backbone, after digestion with *Eco*RI and *Hin*dIII. For TRE-Post-mGRASP-2A-tdTomato, the Post-mGRASP-2A-tdTomato encoding sequence was amplified from pAAV-CAG-Post-mGRASP-2A-tdTomato (#34912, Addgene) by PCR using two oligonucleotide primers containing *Eco*RI and *Hin*dIII restriction sites (5′- caagaattcATGGCACTTCCTAGATGTATG-3′ and 5′-gatAAGCTTACTTATACAGCTCATCC-3′) and inserted into pAAV-TRE backbone, after digestion with *Eco*RI and *Hin*dIII. The plasmids were transformed into stbl2 *E. coli* (#10268019, Invitrogen) grown on Ampicillin (100 μg/ml, #A9518-5G, Sigma)–LB agar (#244520, BD DifcoTM) plates. Positive clones were sequenced (Cosmogenetech, Korea), and the results were analyzed with DNASTAR Navigator (DNASTAR, Madison, WI, USA). The viral particles were produced by the Gene Therapy Center Vector Core at the University of North Carolina at Chapel Hill, Chapel Hill, NC (UNC Vector Core) or by the Viral vector facility of the ETH (Zurich).

### cFostTA-dependent labeling of neurons

The Tet-off system enables to mark cells expressing the IEG *cFos* only when Tetracycline or its analogous Doxycycline (DOX) is absent in the organism. Thus, immediately after viral injection, mice were switched to DOX-containing chow (200 mg. DOX/kg. chow, Bio-Serv) to prevent cFostTA-dependent expression. Three weeks after viral injection we switched the mice to normal chow and on the following day we placed mice in a novel EE for 16 h or performed TFC training. A total of 5 h after exploration of EE or TFC mice were switched back to DOX-containing chow until the time they were sacrificed.

### ArcCre^ERT2^-dependent labeling of neurons

Mice received a single intraperitoneal injection of tamoxifen (175 mg/kg of body weight) right before being placed into the EE or 30’ before TFC training. Tamoxifen was dissolved in 5% of the final volume in 100% Ethanol and further diluted with corn oil to a final concentration of 10 mg/ml. The solution was heated up to 37°C before injection. After exposure to the EE (16 h) or TFC training mice were transferred back into their HC.

### Enriched environment

Enriched environments were created by connecting two rat-cages (37 cm × 60 cm) with an acrylic tunnel (20 cm × 15 cm) resulting in a total area 4,440 cm^2^. Enriched cages contained tunnels, wooden climbing sticks, wooden shelters, running wheels, seesaws, cotton pads, hair curlers, wooden blocks, swinging hammocks, and toys which mice could open and which contained food pellets. Cages also contained a second level connected to the ground floor by a wooden ladder and consisting of a wooden board and climbing ropes allowing mice to reach the lid grit. Food was hidden in the bedding material and spread around the arena to encourage mice to explore the environment.

### Trace fear conditioning

On the training day mice were put into a square conditioning chamber (19 cm × 19 cm, black metal walls, stainless steel grid floor, white light illumination, and ethanol odor) (Panlab) which we defined as Context A. Following 3 min of habituation, mice received 3 pairings of a tone (80 dB, 9 kHz, 20 s duration, CS) and a mild electric foot shock (0.75 mA, 1 s duration, USA) with a trace of 15 s between the tone and the shock and an intra trial interval of 105 s. On probe day mice were tested for their memory recall. To test context memory recall we placed mice into Context A for 3 min. The position of the mouse was tracked automatically and the freezing response was recorded and quantified in real-time with ANY-maze (Stoelting). The amount of freezing was calculated as the percentage of total exploration time during which the mice were immobile. Immobility for more than 250 ms was scored as freezing.

### Histology

We perfused mice intracardially with 1× phosphate-buffered saline (PBS) containing Heparin followed by 4% paraformaldehyde (PFA) in PBS. We then dissected the brains and placed them in 4% PFA in 1× PBS for 24 h, at 4^°^C. Brains were then transferred to 30% sucrose in PBS for 48 h, at 4^°^C. Brain slices (50 μm thick) were prepared with a vibratome (Microm HM 650 V, Thermo Scientific). Slices were permeabilized with 0.2% Triton X-100 in PBS for 1 h and later quenched with 150 mM Glycine in ddH_2_0 for 15 min. Slices were incubated with DAPI (1:1000 in PBS, Thermo Fisher) for 5 min washed with PBS and mounted onto slides with mounting medium (Vectashield).

### Quantification of the fluorescent markers

To quantify the proportion of RFP-, dTomato, or tdTomato-positive dCA1 cells we used a confocal microscope (Zeiss LSM 800) and acquired image stacks (319.28 μm^2^ single section area, 5 μm z-step, 8–10 focal planes) of five representative fields in the dCA1 or dCA3 per mouse using a 40× objective [Zeiss Plan-Apochromat 40×/1.4 NA Oil DIC (UV) VIS-IR]. We acquired DAPI (Thermo Fisher, 405 nm excitation and 465 nm emission wavelengths) or Syto60 (Thermo Fisher, 650 nm excitation and 680 nm emission wavelengths) fluorescence to identify neuronal nuclei and red fluorescence (561 nm excitation and 618 nm emission wavelengths) to identify RFP-, dTomato-, or tdTomato-positive cells. We then manually counted DAPI-positive, Syto60-positive and double positive cells using the ImageJ plugin Cell Counter. When the same neuron was visible in more than one z-slice we only counted it once in the z-plane in which the diameter of the soma was the largest.

To identify GFP-positive puncta we used a confocal microscope (Zeiss LSM 800) and acquired image stacks (319.28 μm^2^ single section area, 1–0.5 μm z-step, 8–30 focal planes) of representative fields in the dCA1 per mouse using a 63× objective [Zeiss Plan-Apochromat 60×/1.5NA Oil DIC (UV) VIS-IR].

### Statistical analysis

For statistics, we used the Kruskal–Wallis test with Dunn’s correction for multiple comparisons, paired Wilcoxon Signed-rank, Mann-Whitney U-test, one-way ANOVA with Sidak’s correction for multiple comparisons and unpaired, two-tailed *t*-test. Prior to performing any statistical test, we tested all distributions to be tested for their likelihood of being Gaussian using the Shapiro–wilk test. We then used non-parametric tests if at least one of the distributions was not Gaussian. Statistical analysis and plotting was done with Prism 8 (GraphPad) software. *p ≤ 0.05, ^**^p ≤ 0.01, ^***^p ≤ 0.001, ^****^p < 0.0001.

## Results

### cFostTA-dependent expression of mGRASP

The promoter of the IEG *cFos* is extensively used to mark engram neurons in mice (Reijmers et al., 2007; Liu et al., 2012; Ramirez et al., 2013, 2015; Denny et al., 2014; Kawashima et al., 2014; Redondo et al., 2014; Tanaka et al., 2014b; Kitamura et al., 2017; DeNardo et al., 2019; Visser et al., 2020; Hwang et al., 2022) and it has recently been used to label synaptic contacts between engram neurons using the eGRASP system (Choi et al., 2018, 2021; Choi and Kaang, 2022). However, it is unclear whether using other transsynaptic GFP complementation systems such as mGRASP, would also enable to label synaptic contacts between engram neurons. We thus tested whether the mGRASP system would yield labeling of synapses under the control of the *cFos* promoter. To this aim, we used a construct in which the *cFos* promoter drives expression of a tTA (Zhang et al., 2015) in combination with two viral constructs in which pre- and post-mGRASP are under the transcriptional control of the Tetracycline Responsive Element (TRE).

### Kinetics of cFostTA-dependent expression in dorsal hippocampal CA1 and CA3

**FIGURE 1:**
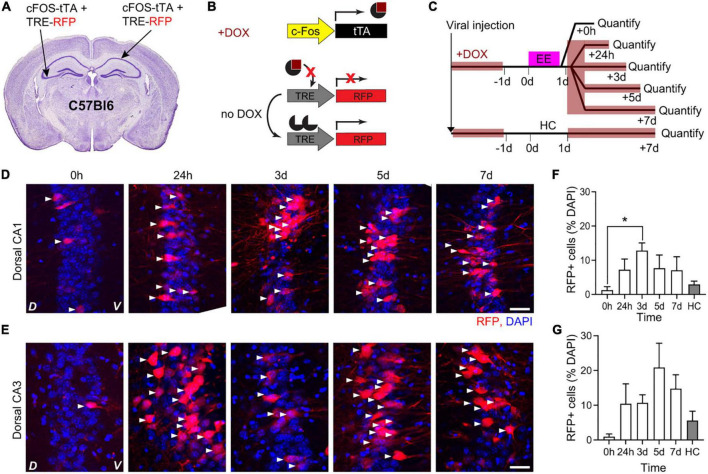
Kinetics of cFostTA-dependent expression of RFP in dorsal hippocampus. Schematic description of the viral injection sites in WT animals **(A)**, the viral constructs injected **(B)**, and the experimental design **(C)**. Confocal pictures, single Z-planes, of dCA1 **(D)** and dCA3 **(E)** at different time points after induction of c-FOS-dependent expression of RFP upon exposure to EE. Red, RFP; blue, DAPI. White triangles indicate RFP-positive cells. D, Dorsal, V, Ventral. Scale bar = 20 μm. **(F)** The percentage of RFP-expressing over DAPI-positive cells in dCA1 at 3 days was significantly higher than 0 h and HC (*p*_0h–24h_ > 0.999, *p_0h–3d_ = 0.02, *p*_0h–5d_ > 0.999, *p*_0h–7d_ > 0.89, *p*_0h–HC_ > 0.99; *n*_0h_ = 5, *n*_24h_ = 5, *n*_3d_ = 7, *n*_5d_ = 3, *n*_7d_ = 5, *n*_HC_ = 5; Kruskal–Wallis test after Dunn’s correction for multiple comparisons). **(G)** The percentages of RFP-expressing over DAPI-positive cells in dCA3 at 3 and 5 days were significantly higher than 0 h when compared directly (*p*_3d–0h_ = 0.019 and *p*_5d–0h_ = 0.035; *n*_3d_ = 7, *n*_0h_ = 5, *n*_HC_ = 5; Mann–Whitney U-test) but showed only a trend after correction for multiple comparisons (*p* = 0.076; Kruskal–Wallis test after Dunn’s correction for multiple comparisons).

The tTA system leads to transient *cFos*-dependent gene expression and synaptic mGRASP should be most evident at the peak of cFostTA-dependent gene expression. We thus quantified the kinetics of cFostTA-dependent gene expression in dorsal hippocampal CA1 and CA3 (dCA1 and dCA3, respectively) upon exploration of an EE. To this aim, we injected the right dCA1 and left dCA3 of C57Bl6 animals each with two AAVs encoding for cFostTA and TRE-RFP (Red Fluorescent Protein), respectively (Figures 1A, B). Immediately after viral transduction, we switched the mice to DOX-containing food to prevent cFostTA-dependent RFP expression. Three weeks after viral transduction we switched the mice to normal chow to enable RFP expression and on the following day we placed five groups of mice in EE for 16 h (Figure 1C). After EE, we switched back the mice to DOX-containing food. We sacrificed different groups at different time points after induction and processed brain slices for confocal microscopy to quantify cFostTA-dependent RFP appearance in dCA1 and dCA3 (Figures 1D, E). One additional group of mice was housed in their Home Cage (HC) and served as a control for baseline expression (Figure 1C). In dCA1 the percentage of RFP-positive cells peaked at 3 days after induction (Figure 1F). In dCA3 the percentage of RFP-positive cells at 3 and 5 days post induction was significantly higher than 0 h only by pairwise comparison (Figure 1G). Altogether, these data show a trend toward peak expression for cFostTA-dependent RFP between 3 and 5 days after induction in both dCA1 and dCA3.

### cFostTA-dependent expression of mGRASP upon exposure to enriched environment or trace fear conditioning labels a subset of dCA1 cells without apparent GFP reconstitution

**FIGURE 2:**
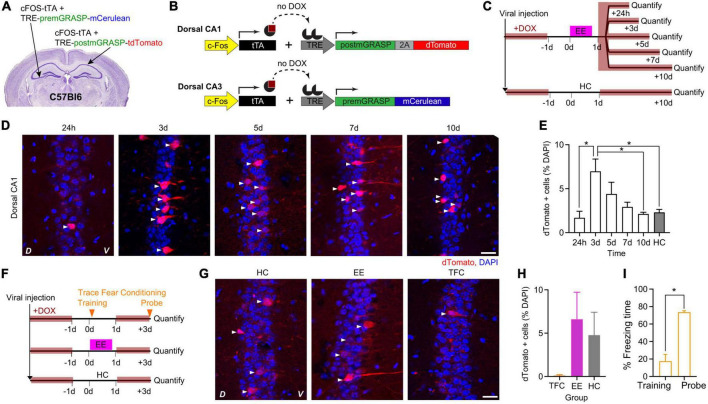
cFostTA-dependent expression of mGRASP in dCA1 upon exposure to an Enriched Environment or Trace Fear Conditioning. Schematic description of the viral injection sites in WT animals **(A)**, the viral constructs injected **(B)** and the experimental design **(C)** to determine the expression kinetics of post-mGRASP in dCA1 under the control of cFos upon exposure to EE or HC. **(D)** Confocal pictures, single Z-planes, of dCA1 at different time points after induction of post-mGRASP expression. Red, dTomato; blue, DAPI. White triangles indicate RFP-positive cells. D, Dorsal, V, Ventral. Scale bar = 20 μm. **(E)** Percentage of dTomato-expressing over DAPI-positive cells in dCA1 after exposure to EE or HC. The levels of dTomato reached a peak at 3 days (*p*_3d–24h_ = 0.01, *p*_3d–5d_ = 0.7, *p*_3d–7d_ = 0.3, *p*_3d–10d_ = 0.037, *p*_3d–HC_ = 0.048; all *n* = 5; Kruskal–Wallis test after Dunn’s correction for multiple comparisons). **(F)** Schematic description of the experimental design to determine the dCA1 expression levels of post-mGRASP under the control of cFos upon exposure to TFC, EE, or HC. **(G)** Confocal pictures, single Z-planes, of dCA1 at different time points after induction of post-mGRASP. Red, dTomato; blue, DAPI. White triangles indicate dTomato-positive cells. D, Dorsal, V, Ventral. Scale bar = 20 μm. **(H)** The percentage of dTomato-expressing over DAPI-positive cells in dCA1 was not significantly different after exposure to TFC, EE or HC (*p*_TFC–HC_ = 0.41 and *p*_EE–HC_ = 0.26, all *n* = 6; Kruskal–Wallis test after Dunn’s correction for multiple comparisons). **(I)** Percentage of freezing time during the context probe 3 days after TFC training (*p* = 0.03, *n* = 6; Wilcoxon Signed-rank test). *p < 0.05.

To characterize the peak of cFostTA-dependent mGRASP expression, we quantified the kinetics of dTomato expression in the dCA1 upon exposure to EE. To this aim, we injected the right dCA1 of C57Bl6 mice with two AAVs encoding for cFostTA and TRE-post-mGRASP and the left dCA3 of the same animals with two AAVs encoding for cFostTA and TRE-pre-mGRASP (Figures 2A, B) and switched the mice to DOX-containing food. Three weeks after viral transduction we switched to normal chow and on the following day we placed mice in a novel EE for 16 h (Figure 2C). After EE, we switched the mice back to DOX-containing food. We sacrificed each group at a different time point after induction to quantify *cFos*-dependent dTomato appearance in dCA1 (Figure 2D). One group of mice was housed in their Home Cage (HC) and served as a control for baseline expression (Figure 2C). In dCA1 the percentage of dTomato-positive cells peaked at 3 days after induction (Figures 2D, E), consistently with the previous experiment. We also detected mCerulean-positive cells in the contralateral CA3 at 3 days after induction (Supplementary Figures 1A, B). However, we could not detect any GFP reconstitution on dendrites or somas of dTomato-expressing dCA1 neurons (Supplementary Figure 1C).

Next, we investigated the differences in cFostTA-driven dTomato induction upon exploration of an EE in comparison to learning of the hippocampal-dependent learning task Trace Fear Conditioning (TFC). To this aim we injected the brains of WT mice with AAVs and fed the mice with DOX-containing food as in the previous experiment (Figures 2A, B). Three weeks after viral transduction we switched to normal chow and on the following day one group of mice underwent TFC, another group of mice explored an EE for 16 h and a third group of mice was housed in HC and served as a control group (Figure 2F). The dTomato expression levels of the EE and HC mice were consistent with the previous experiment. However, we found virtually no dTomato-positive cell after TFC with only 1 out of 5 mice showing a single cell labeled in a single field of view (Figures 2G, H), despite a significant recall of the association between context and shock (Figure 2I). Under these conditions we could not detect any GFP reconstitution on dTomato-expressing dCA1 neurons.

### ArcCre^ERT2^-dependent labeling of synaptic engrams

The promoter of the IEG *Arc* has also been used to mark engram neurons in mice (Kawashima et al., 2009, 2013; Denny et al., 2014; Attardo et al., 2018; Castello-Waldow et al., 2020) but it has not been used to label synaptic contacts between engram neurons. We thus tested whether the mGRASP system could work under the control of *Arc* promotor. To this aim, we employed a transgenic mouse line previously used to label neurons active during a defined time window (Guenthner et al., 2013; Castello-Waldow et al., 2020). As in this line the promoter of the Arc gene drives expression of a Cre recombinase gated by Tamoxifen (TAM), we used viral constructs in which pre- and post-mGRASP were under the transcriptional control of Cre (Kim et al., 2011).

### Kinetics of ArcCre^ERT2^ -dependent expression in hippocampal dCA1 and dCA3

**FIGURE 3:**
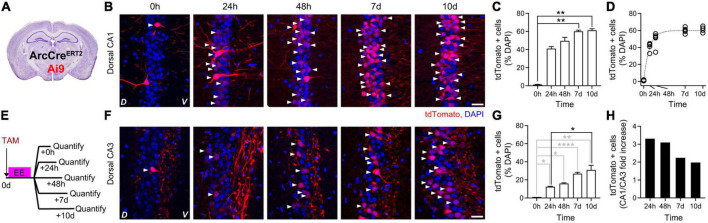
Kinetics of ArcCre^ERT2^-dependent expression of tdTomato in dorsal hippocampus upon exposure to an Enriched Environment. **(A)** Schematic description of ArcCre^ERT2^—Ai9 double transgenic animals. **(B)** Confocal pictures, single Z-planes, of dCA1 at different time points after induction of tdTomato expression. Red, tdTomato; blue, DAPI. White triangles indicate tdTomato-positive cells. D, Dorsal, V, Ventral. Scale bar = 20 μm. Modified from Castello-Waldow et al. (2020). **(C)** Percentage of tdTomato-expressing over DAPI-positive cells in dCA1 at different time points after exposure to EE. The levels of tdTomato at 7 and 10 days were significantly higher than baseline (0 h) (*p*_7d–0h_ = 0.0012 and *p*_10d–0h_ = 0.0007, all other *p* > 0.14, all *n* = 5; Kruskal–Wallis test after Dunn’s correction for multiple comparisons). Modified from Castello-Waldow et al. (2020). **(D)** Single exponential fit to the time course of ArcCreER^T2^-dependent tdTomato expression shown in C. Plateau = 60%, *R*^2^ = 0.96. Circles represent single datapoints (percentage of tdTomato-expressing over DAPI-positive cells in dCA1 at different time points after exposure to EE per mouse), dashed line is the best fit curve. **(E)** Schematic description of the experimental design to determine the expression kinetics of tdTomato in dCA3 under the control of endogenous Arc upon exposure to EE. **(F)** Confocal pictures, single Z-planes, of dCA3 at different time points after induction of tdTomato expression. Red, tdTomato; blue, DAPI. White triangles indicate tdTomato-positive cells. D, Dorsal, V, Ventral. Scale bar = 20 μm. **(G)** Percentage of tdTomato-expressing over DAPI-positive cells in dCA1 at different time points after exposure to EE. The levels of tdTomato were significantly higher than baseline (0 h) on all days (*p*_24–0h_ = 0.03, *p*_48h–0h_ = 0.03, *p*_7d–0h_ < 0.0001, *p*_10d–0h_ = 0.0091; *n*_0h_ = 1, *n*_24h_ = 2, *n*_48h_ = 2, *n*_7d_ = 5, *n*_10d_ = 4; One-sample *t*-test). Comparison to the 24 h timepoint retrieved a trend at time point 7 days and a significant increase at time point 10 (*p*_48h–24h_ = 0.93, *p*_7d–24h_ = 0.0719, *p*_10d–24h_ = 0.025; *n*_24h_ = 2, *n*_48h_ = 2, *n*_7d_ = 5, *n*_10d_ = 4; One-way ANOVA with Sidak correction for multiple comparisons). **(H)** Ratio of dCA1 over dCA3 tdTomato expression levels at different time points after induction. *p < 0.05, **p < 0.01, ****p < 0.0001.

To identify the peak of *Arc*-dependent expression, we quantified the kinetics of tdTomato appearance in the hippocampal dCA1 and dCA3 upon exposure to EE. To this aim, we crossed the ArcCre^ERT2^ transgenic mouse line with the Ai9 transgenic mouse line to obtain a double transgenic line (ArcCre^ERT2^-Ai9) in which the onset of *Arc*-dependent tdTomato expression was gated by TAM injection (Figure 3A). We had previously determined the appearance kinetics upon a 16 h-long exposure to an EE in the dCA1 and found a significant increase in tdTomato-positive cells plateauing at 7 days after induction [Figures 3B–D, modified from Castello-Waldow et al. (2020)]. Now, we used five additional groups of the ArcCre^ERT2^-Ai9 double transgenic mice to perform the same quantification in the dCA3. We injected a single dose of TAM intraperitoneally in all groups right before exploration of EE and quantified tdTomato appearance at different time points (Figures 3E, F). The number of tdTomato-positive cells was higher than 0 at all time points, from 24 h to 10 days, with peak expression at 10 days after induction (Figure 3G). Expression levels in dCA1 were higher than in dCA3 within the 10 days time window (Figure 3H). Altogether, these data show a peak expression for ArcCre^ERT2^-driven tdTomato between 7 and 10 days after induction in both dCA1 and dCA3.

### *Arc*-dependent expression of mGRASP upon exposure to enriched environment or trace fear conditioning labels a subset of dCA1 cells and yields GFP reconstitution

**FIGURE 4:**
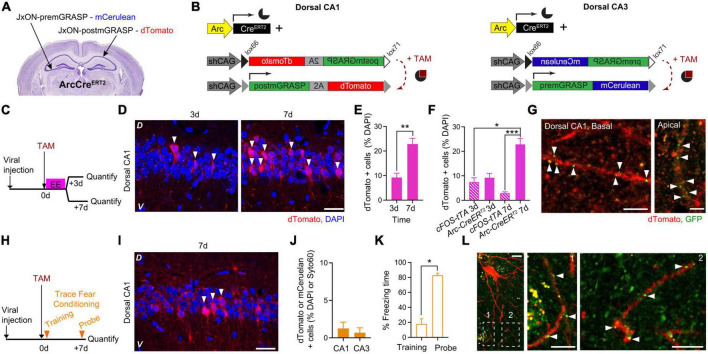
ArcCre^ERT2^-dependent expression of mGRASP in dCA1 upon exposure to an Enriched Environment or Trace Fear Conditioning. Schematic description of the viral injection sites in ArcCre^ERT2^ transgenic animals **(A)** and the viral constructs injected **(B)**. **(C)** Experimental design to determine the expression levels of post-mGRASP in dCA1 under the control of Arc at two different time points after exposure to EE. **(D)** Confocal pictures, single Z-planes, of dCA1 at different time points after induction of post-mGRASP expression. Red, dTomato; blue, DAPI. White triangles indicate dTomato-positive cells. D, Dorsal, V, Ventral. Scale bar = 20 μm. **(E)** Percentage of dTomato-expressing over DAPI-positive cells in dCA1 3 and 7 days after exposure to EE. The level of dTomato at 7 days was significantly higher than at 3 days (*p* = 0.0095; *n*_3_ = 4, *n*_7_ = 6; Mann–Whitney U-test). **(F)** Percentage of dTomato-expressing over DAPI-positive cells in dCA1 3 and 7 days after exposure to EE in the cFostTA (striped) or ArcCre^ERT2^ (solid) systems. The level of dTomato at 7 days was significantly higher in the ArcCre^ERT2^ system than at 3 and 7 days in the cFostTA system (*p*_7dArc7dFOS_ = 0.0005, *p*_7dArc3dFOS_ = 0.017, all other *p* > 0.31; *n*_7dArc_ = 6, *n*_7dFOS_ = 5, *n*_3dArc_ = 4, *n*_3dFOS_ = 10; Kruskal–Wallis test after Dunn’s correction for multiple comparisons). **(G)** Confocal pictures, single Z-planes, of apical (right) and basal (left) dendritic segments of dCA1 pyramidal neurons at 7 days after induction of post-mGRASP expression showing GFP-fluorescent puncta colocalizing with dTomato-positive dendritic segments. Red, dTomato; green, GFP. White triangles indicate synaptic GFP reconstitution. D, Dorsal, V, Ventral. Scale bars = 5 μm. **(H)** Experimental design to determine the dCA1 and dCA3 expression levels of post-mGRASP under the control of Arc after TFC. **(I)** Confocal picture, single Z-plane, of dCA1 7 days after induction of post-mGRASP expression. Red, dTomato; blue, DAPI. White triangles indicate dTomato-positive cells. D, Dorsal, V, Ventral. Scale bar = 20 μm. **(J)** Percentage of dTomato- (dCA1) or mCerulean- (dCA3) expressing over DAPI- (dCA1) or Syto60- (dCA3) positive cells 7 days after exposure to FC. **(K)** Percentage of freezing time during the context probe 7 days after TFC training (*p* = 0.015, *n* = 7; Wilcoxon Signed-rank test). **(L)** Confocal pictures, single Z-planes, of a dCA1 pyramidal neuron and dendritic segments (insets) 7 days after induction of post-mGRASP expression showing GFP-fluorescent puncta colocalizing with dTomato-positive dendritic segments. Red, dTomato; green, GFP. White triangles indicate synaptic GFP reconstitution. Inset number 2 is rotated 90 degrees from the original orientation. Scale bars: left = 10 μm, middle and right = 5 μm. *p < 0.05, **p < 0.01, ***p < 0.001.

While cFostTA-dependent dTomato expression peaked at 3 days after induction (Figure 2E), ArcCreER^T2^-dependent labeling followed longer time scales (Figure 3C). Thus to compare cFostTA- *versus* ArcCre^ERT2^-dependent mGRASP expressions, we quantified ArcCre^ERT2^-dependent dTomato appearance in the hippocampal dCA1 at an earlier (3 days) and a later (7 days) time points after overnight exposure to EE. To this aim, we injected the right dCA1 and the left dCA3 of two groups of ArcCre^ERT2^ mice with AAVs encoding for Cre-dependent-post-mGRASP and Cre-dependent-pre-mGRASP, respectively (Figures 4A, B). Three weeks after viral transduction we injected a single dose of TAM (175 mg/kg) intraperitoneally in all groups right before exploration of EE and quantified ArcCre^ERT2^-dependent dTomato appearance 3 or 7 days later (Figure 4C). We detected dTomato-positive cells in dCA1 (Figure 4D) and the percentage of dTomato-expressing cells was higher at 7 days than at 3 days after induction (Figure 4E). Interestingly, while the proportion of cells positive for cFostTA- and ArcCre^ERT2^-dependent dTomato was similar at 3 days after induction (7.5 and 9.2% of all DAPI cells, respectively), at 7 days the number of cells positive for ArcCre^ERT2^-dependent dTomato was significantly higher than the number of cells positive for cFostTA-dependent dTomato (3 and 23% of all DAPI cells, respectively) (Figure 4F).

Importantly, at 7 days–but not at 3 days–after induction we detected GFP reconstitution on approximately 40% of apical and basal dendrites of dTomato dCA1 neurons (Figure 4G and Supplementary Figures 2A, B).

Finally, we tested whether the ArcCre^ERT2^ system would also be able to visualize synaptic engrams after hippocampal-dependent learning. To this aim, we transduced another group of ArcCre^ERT2^ mice as in the previous experiment, 3 weeks after viral transduction we injected a single dose of TAM intraperitoneally right before TFC training and quantified the number of dTomato-positive cells 7 days later (Figure 4H). We detected dTomato expression in dCA1 7 days after induction (Figure 4I). dTomato expression in dCA1 and mCerulean expression in dCA3 was limited to a very small fraction of cells (Figure 4J), despite a significant recall of the association between context and shock (Figure 4K). In a single instance, we detected GFP reconstitution on a small subset of dendrites of dTomato-expressing dCA1 neurons at 7 days after TFC training (Figure 4L).

## Discussion

We used two different genetic schemes—based on the expression of the IEGs cFos and Arc—to express the virally encoded GFP transsynaptic complementation system mGRASP (Kim et al., 2011), with the aim to label structural synaptic engrams in the hippocampal dCA1 of mice. While both schemes are based on activity-dependent transcription, they show important differences. In the cFostTA-based scheme, the promoter of *cFos* is encoded in a viral construct and controls (virally transduced) mGRASP expression through the tTA-TRE system, while in the ArcCre^ERT2^-based scheme the promoter of *Arc* is endogenous to the genome and controls (virally transduced) mGRASP expression through the Cre-Lox system. These differences affect the expression kinetics of the reporters and thus (i) the kinetics of mGRASP-positive cells appearance, (ii) the number of mGRASP-positive cells, and (iii) the detection of transsynaptic GFP reconstitution.

### cFostTA and ArcCreER^T2^ systems display different expression kinetics

Appearance of cFostTA-dependent expression peaked at approximately 3 days after induction by exploration of an EE, with the number of dTomato-positive cells 10 days after induction being statistically indistinguishable from baseline in mice transduced with the TRE-post-mGRASP construct. This is because the cFostTA system triggers only transient expression of the reporter as the tTA drives transcription only during the time window where DOX is not present. Thus, after the induction peak, dTomato is degraded and its expression levels fall to baseline. In contrast, in the ArcCre^ERT2^ system the number of tdTomato-positive cells increased and reached plateau at 7 days after induction. This is because the ArcCre^ERT2^ system triggers transcription when TAM is present and expresses the reporter constitutively after that. Thus, after the induction peak, dTomato keeps being replenished and its expression levels plateau rather than falling back to baseline.

### The ArcCreER^T2^ system labels more dCA1 cells than the cFostTA system

At peak expression after EE exploration—3 days for the cFos and 7 days for Arc system—we detected almost three times as many ArcCre^ERT2^- as cFostTA-dTomato positive cells. Although, the IEGs cFos and Arc mark non-fully overlapping neuronal populations, their mRNAs are found in a similar proportion of cells upon activation (Guzowski et al., 2001; Miyashita et al., 2009). Thus, the difference we observe must be due to difference between the genetic systems we employ rather than the IEG promoters. In particular, we think that the higher accumulation of ArcCre^ERT2^-dependent dTomato in combination with the detection threshold of dTomato fluorescence at the single-cell level might explain the difference in numbers between ArcCre^ERT2^- and cFostTA-dTomato positive cells. Arc expression is not uniform, with active cells expressing the IEG at different levels upon induction (Attardo et al., 2018). As such, also the expression of Arc-driven Cre^ERT2^ is bound not to be uniform, with some cells expressing higher and some others lower levels of Cre^ERT2^. As the mGRASP is virally transduced, each cell contains multiple copies of the DNA encoding for dTomato, hence the limiting factor for dTomato production is likely the amount of Cre^ERT2^. Higher-Cre^ERT2^-expressing cells will be able to produce amounts of dTomato sufficient to cross the threshold of detectability earlier, while lower-expressing cells will require longer accumulation of dTomato in order to cross the threshold for detectability. Importantly, this buildup is absent in the cFostTA system as tTA-dependent transcription is switched off by DOX administration, thus lower-cFostTA-expressing cells do not have the time to accumulate enough dTomato to cross the detectability threshold. Hence, in the cFostTA system at its expression peak only the subset of higher-cFostTA-expressing cells will be detectable as dTomato-positive. In contrast, a larger number of ArcCre^ERT2^-expressing cells—including lower- and higher-expressing cells—will be detectable as dTomato-positive in the ArcCre^ERT2^ system at its expression peak. In other words, the cFostTA system works as a high-pass filter over the *cFos* expression range while the ArcCre^ERT2^ system integrates over the *Arc* expression range.

### Transsynaptic GFP reconstitution in the ArcCreER^T2^ but not in the cFostTA system

As a consequence of the integrative process in the ArcCre^ERT2^ system, higher-ArcCre^ERT2^-expressing cells at 7 days will also accumulate a greater amount of GFP in comparison to higher-cFostTA-expressing cells at 3 days. This explains why we detected GFP reconstitution only in the ArcCre^ERT2^—but not the cFostTA—system and only at 7 days—but not at 3 days—after induction. Further protein buildup in the ArcCre^ERT2^ system might be necessary to achieve an amount of GFP sufficient to cross the threshold for detectability or even possibly for transsynaptic reconstitution. Recent work did, however, report GFP reconstitution in the dual eGRASP system under the control of cFostTA (Choi et al., 2018, 2021). This discrepancy is most likely due to the fact that in the aforementioned study the split GFP was engineered with an additional S72A mutation and to take advantage of the stronger interaction between peptide p40 and the SH3 domain in the post-eGRASP construct to enhance fluorescence (Choi et al., 2018), thus using the eGRASP system might lead to a brighter labeling.

The level of ArcCre^ERT2^-driven accumulation of mGRASP proteins in single cells did not depend on the behavioral stimulus that triggered cellular activity, as we detected GFP reconstitution in dCA1 after exploration of EE as well as after TFC training. However, TFC training marked a smaller subset of cells than exploration of an EE in dCA1 and dCA3 of ArcCre^ERT2^ mice [this work Figures 4E, J, and (Castello-Waldow et al., 2020)] and higher sparseness of pre- and postsynaptic mGRASP-expressing cells makes detection of GFP puncta much less likely. This explains why we detected GFP reconstitution after TFC training only very sporadically.

### Enduring structural connectivity between dCA3 and CA1 Arc-expressing cells upon learning

The observation of mGRASP *trans* synaptic reconstitution between dCA3 and dCA1 Arc-positive cells indicates that structural connectivity between Arc-expressing neuronal ensembles is present 1 week after memory encoding. This result is consistent with the hypothesis that enduring synaptic connectivity can re-entrain a memory state days after memory acquisition Josselyn and Tonegawa, 2020). However, as hippocampal CA1 excitatory synaptic network is highly dynamic (Attardo et al., 2015; Pfeiffer et al., 2018) and the Arc-based system is always driving transcription of mGRASP after induction, we cannot distinguish between synapses that were present at memory acquisition and synapses that were added at a later time point. Importantly, as mGRASP expression in the ArcCre^ERT2^-dependent system provides long term labeling of synaptic engrams, it could be combined with longitudinal intravital optical imaging to tackle this issue. In fact, this combination would enable not only to detect the presence but also to track the persistence of synaptic engrams and thus to follow the temporal evolution of synaptic engrams in live mice over several weeks; thereby, enabling to investigate the function of synaptic engrams in the face of continuous learning.

## Data availability statement

The raw data supporting the conclusions of this article will be made available by the authors, without undue reservation.

## Ethics statement

This animal study was reviewed and approved by Regierung von Oberbayern – Veterinärwesen.

## Author contributions

BM and SS performed the majority of the experiments and analyzed the data. AU, TC-W, and RH performed a subset of the experiments. AC and AA procured funding. AU and AA designed the experiments. AU, HK, NH, and AA supervised the project. AA wrote the manuscript. All authors contributed to the article and approved the submitted version.

## Supplementary material

Supplementary Figure 1 cFostTA-dependent expression of mCerulean in dCA3 and of dTomato in dCA1 without GFP reconstitution. **(A)** Schematic description of the viral constructs injected. **(B)** Confocal picture, single Z-plane, of dCA3 3 days after induction of c-FOS-dependent expression of mCerulean upon exposure to EE. Red, Syto60; Cyan, mCerulean. White triangles indicate mCerulean-positive cells. L, Lateral, M, Medial. Scale bar = 10 μm. **(C)** Confocal picture, Maximum Intensity Projection of 5 Z-plane, of dCA1 3 days after induction of c-FOS-dependent expression of mCerulean upon exposure to EE. Red, dTomato; Green, GFP. Scale bar = 7 μm.

Supplementary Figure 2 A subset of dTomato + dCA1 dendrites show GFP reconstitution. **(A)** Schematic description of the viral constructs injected. **(B)** The percentage of apical and basal dTomato-expressing dCA1 dendrites showing GFP reconstitution was not significantly different (*p* = 0.45; *n*_Apical_ = 7, *n*_Basal_ = 5, image stacks; unpaired *t*-test).
