# Supplementary material for: Arc-driven mGRASP highlights CA1 to CA3 synaptic engrams
Source: Front Behav Neurosci. 2023 Jan 30;16:1072571. doi: 10.3389/fnbeh.2022.1072571 (PMC9924068; doi:10.3389/fnbeh.2022.1072571)
Supplement: Supplementary file 1 [file Table_1.docx]

**Arc-driven mGRASP highlights CA1 to CA3 synaptic engrams**

Murthy K.B.B.^1,2^*, Somatakis S.^3^*, Ulivi A.F.^1^, Klimmt H.^1,4^, Castello-Waldow T.P.^3^, Haynes N.^3^, Huettl R.E.^3^, Chen A.^4,5^ and Attardo A.^1,2,4^

^1^ Leibniz Institute for Neurobiology, Magdeburg, Germany

^2^ Graduate School of Systemic Neuroscience, Munich, Germany

^3^ Max Planck Institute of Psychiatry, Munich, Germany

^4^ International Max Planck Research School for Translational Psychiatry, Munich, Germany

^5^ Weizmann Institute of Science, Rehovot, Israel

* These authors contributed equally to this work and share first authorship

Correspondence to: alessio.attardo@lin-magdeburg.de

**Supplementary Table 1** | **Origin and titers of the viruses used in this work**
