# Supplementary figures and images for: Arc-driven mGRASP highlights CA1 to CA3 synaptic engrams
Source: Front Behav Neurosci. 2023 Jan 30;16:1072571. doi: 10.3389/fnbeh.2022.1072571 (PMC9924068; doi:10.3389/fnbeh.2022.1072571)

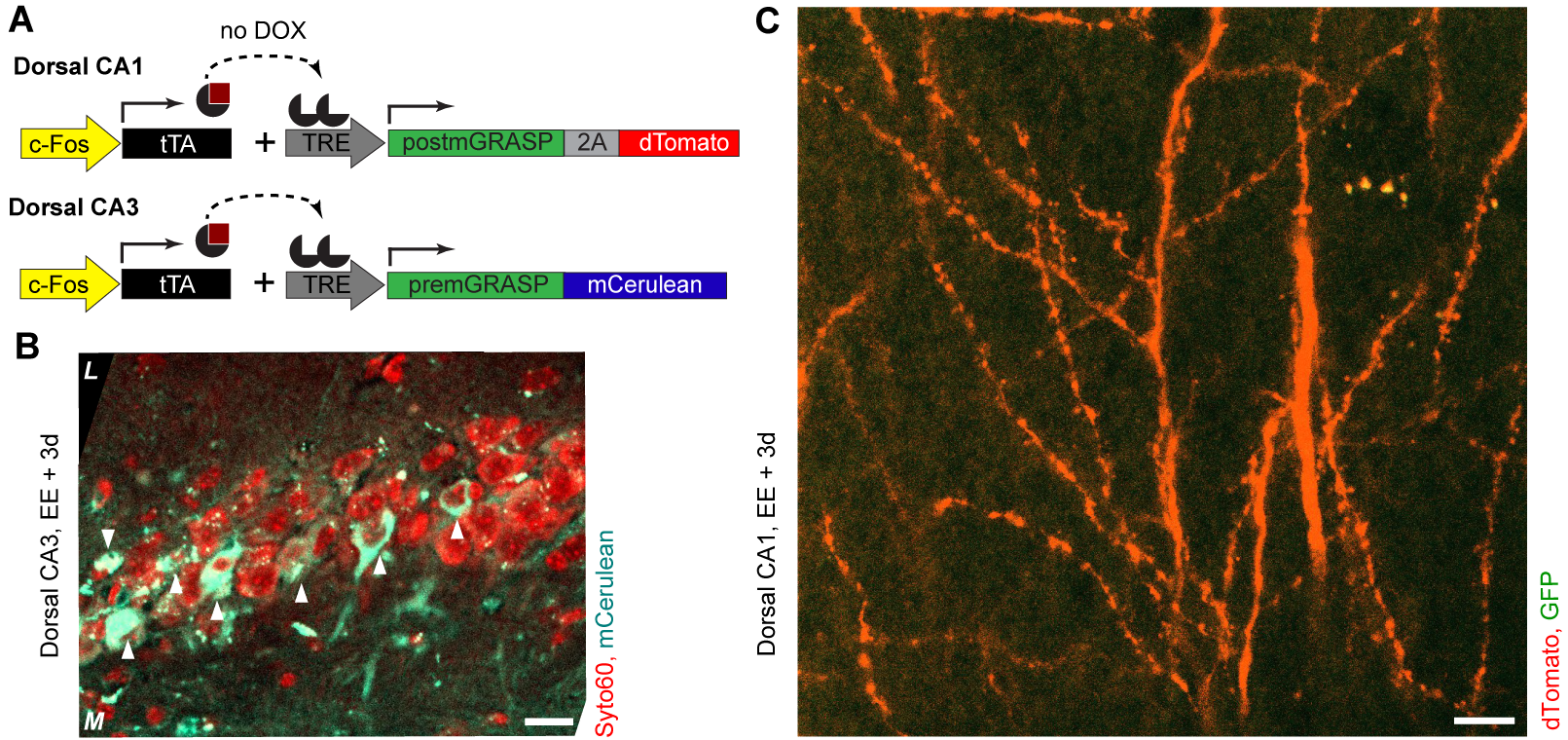

Supplement: Supplementary Figure 1 — cFostTA-dependent expression of mCerulean in dCA3 and of dTomato in dCA1 without GFP reconstitution. (A) Schematic description of the viral constructs injected. (B) Confocal picture, single Z-plane, of dCA3 3 days after induction of c-FOS-dependent expression of mCerulean upon exposure to EE. Red, Syto60; Cyan, mCerulean. White triangles indicate mCerulean-positive cells. L, Lateral, M, Medial. Scale bar = 10 μm. (C) Confocal picture, Maximum Intensity Projection of 5 Z-plane, of dCA1 3 days after induction of c-FOS-dependent expression of mCerulean upon exposure to EE. Red, dTomato; Green, GFP. Scale bar = 7 μm. [file Image_1.TIF]

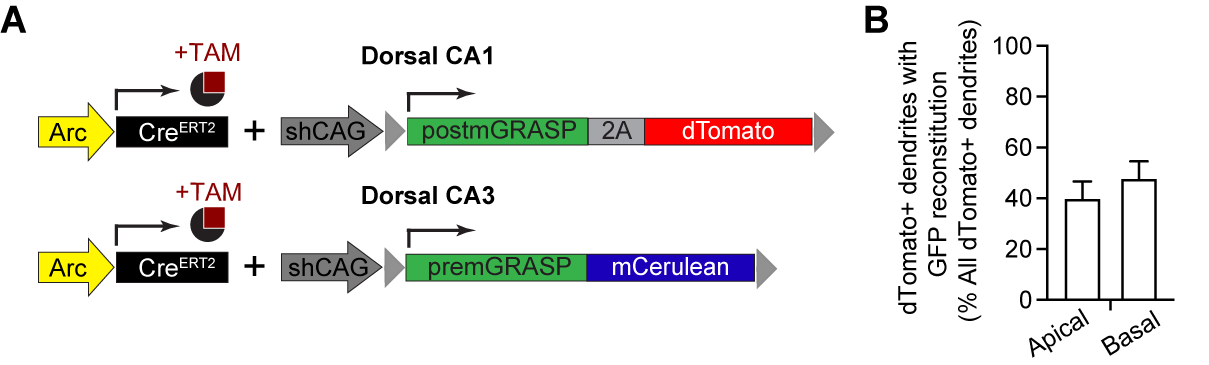

Supplement: Supplementary Figure 2 — A subset of dTomato + dCA1 dendrites show GFP reconstitution. (A) Schematic description of the viral constructs injected. (B) The percentage of apical and basal dTomato-expressing dCA1 dendrites showing GFP reconstitution was not significantly different (p = 0.45; nApical = 7, nBasal = 5, image stacks; unpaired t-test). [file Image_2.TIF]
